# Blood Collection from the American Horseshoe Crab, Limulus Polyphemus

**DOI:** 10.3791/958

**Published:** 2008-10-13

**Authors:** Peter Armstrong, Mara Conrad

**Affiliations:** Department of Molecular and Cell Biology, University of California, Davis; Marine Biological Laboratory - MBL; Department of Biological Sciences, Hunter College of CUNY

## Abstract

The horseshoe crab has the best-characterized immune system of any long-lived invertebrate.  The study of immunity in horseshoe crabs has been facilitated by the ease in collecting large volumes of blood and from the simplicity of the blood.  Horseshoe crabs show only a single cell type in the general circulation, the granular amebocyte.  The plasma has the salt content of sea water and only three abundant proteins, hemocyanin, the respiratory protein, the C-reactive proteins, which function in the cytolytic destruction of foreign cells, including bacterial cells, and α2-macroglobulin, which inhibits the proteases of invading pathogens.  Blood is collected by direct cardiac puncture under conditions that minimize contamination by lipopolysaccharide (a.k.a., endotoxin, LPS), a product of the Gram-negative bacteria.  A large animal can yield 200 - 400 mL of blood.  For the study of the plasma, blood cells are immediately removed from the plasma by centrifugation and the plasma can then be fractionated into its constituent proteins.  The blood cells are conveniently studied microscopically by collecting small volumes of blood into LPS-free isotonic saline (0.5 M NaCl) under conditions that permit direct microscopic examination by placing one of more LPS-free coverglasses on the culture dish surface, then mounting those coverglasses in simple observation chambers following cell attachment.  A second preparation for direct observation is to collect 3 - 5 mL of blood in a LPS-free embryo dish and then explanting fragments of aggregated amebocytes to a chamber that sandwiches the tissue between a slide and a coverglass.  In this preparation, the motile amebocytes migrate onto the coverglass surface, where they can readily be observed.  The blood clotting system involves aggregation of amebocytes and the formation of an extracellular clot of a protein, coagulin, which is released from the secretory granules of the blood cells.  Biochemical analysis of washed blood cells requires that aggregation and degranulation does not occur, which can be accomplished by collecting blood into 0.1 volumes of 2% Tween-20, 0.5 M LPS-free NaCl, followed by centrifugation of the cells and washing with 0.5 M NaCl.

**Figure:**
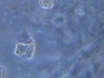


## Protocol

### Anatomical features of the horseshoe crab relevant to bleeding (Fig. 1)


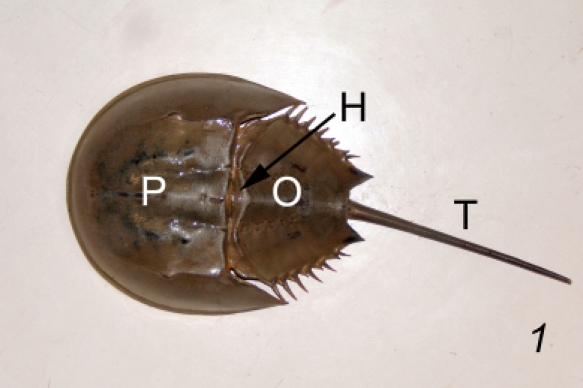


**Figure 1**

The three major divisions of the body, from anterior to posterior, are the prosoma (P), the opisthosoma (O) , and the telson (T) ^1^.
The anterior and lateral free margins of the prosoma is the flange.
The posterior-most indentation of the opisthosoma, where the telson articulates, is the terminal bay.
The joint where the prosoma and the opisthosoma articulate is the hinge (H).
The heart is located along the dorsal midline, just beneath the carapace of prostoma and opisthosoma^2 ^(Fig. 2).
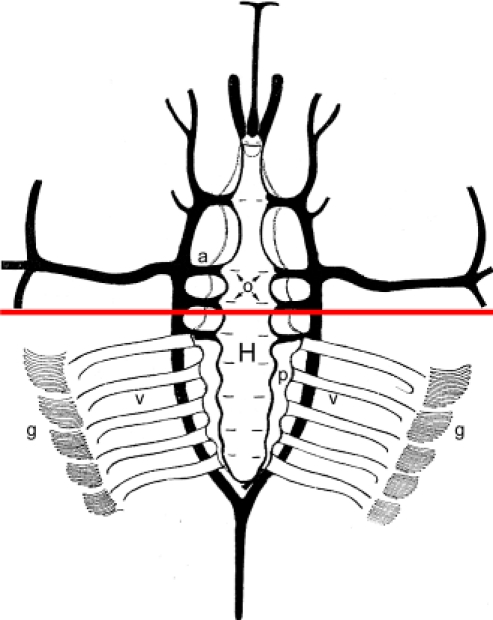
**Figure 2**

### General considerations, sterility and protection from exposure to lipopolysaccharide (endotoxin)

The major enemy of the successful bleed is cell clumping, exocytosis, and formation of the coagulin clot. Cell aggregation and exocytosis are stimulated by lipopolysaccharide (a.k.a., endotoxin, LPS), a product of the Gram-negative bacteria. The threshold concentration of LPS for exocytosis for washed cells is 0.1 - 1 mg/mL but cells suspended in plasma are activated by significantly lower concentrations ^3^. Endotoxin is not inactivated by simple autoclaving and can be assumed to be present on surfaces and in solutions and reagents that are not certified to be endotoxin-free.
To reduce the chances of clotting during collection of blood, select only perfect, undamaged animals for bleeding. Pre-chill the animals 1-2 h in the 4°C room. Practice sterile technique. Avoid contamination with endotoxin.
LPS-free 3% saline is used in clinical medicine and can be purchased from medical suppliers (see table of specialized reagents and supplies). LPS can be removed from glassware and metal by incubation at 180°C for 4 hours. Sterile single-use syringe needles, Petri-style culture dishes, and screw-cap centrifuge tubes are all LPS-free and can be used without modification so long as proper sterile technique is practiced.
The stability of the blood cells of different animals differs, with the cells of some individual animals undergoing spontaneous degranulation, resulting in the formation of the coagulin clot, even when the animal is bled with the greatest care. The blood cells of other individual horseshoe crabs are significantly more stable and, when proper procedures are followed, remain fully granular during bleeding and separation of cells from plasma.
Several agents have been reported to stabilize the blood cells following bleeding, including caffeine (bleed into 0.25 volumes of 10 mM caffeine in LPS-free 3% NaCl) ^4^, propranolol (1 mM final concentration) ^5^, dimethylsulfoxide ^6^, divalent cation chelation (bleed into an equal volume of 0.1 M dextrose, 0.03 M sodium citrate, 0.026 M citric acid, 10 mM disodium ethylenediaminetetraacetic acid (Na_2_EDTA), pH 4.6) ^7^, inhibitors of the G-protein and phospholipase-C pathways (cholera and pertussus toxins, U73122) ^8^, substitution of chloride with isethionate anion ^9^, chloride channel blockers ^9^, cyclic-AMP antagonists ^9^, sulfhydryl reagents (5 mM N-ethyl maleimide, NEM) ^5^, and the membrane-active detergent Tween-20 (bleed into 0.1 volume 2% Tween-20 in LPS-free 0.5 M NaCl).

### Supplies and reagents for bleeding the horseshoe crab: collection of large volumes of blood

one or more large, undamaged horseshoe crab
squirt bottle containing 70% ethanol
Kimwipes
14 gauge needle
sturdy forceps
ice bucket with ice
50 mL screw-cap plastic disposable centrifuge tubes
rack to hold the 50 mL tubes
counter-top centrifuge
sterile 50 mL serological pipettes
bulb-type pipette filler
disposable sterile filter apparatus
vacuum pump
LPS-free 3% NaCl solution (for collection of blood cells)
Tween-20 (for collection of blood cells)

#### Preparations for large-volume bleeding

For bleeding, use large, undamaged animals. Chill the animal for 1 h in a cold room before bleeding.
Establish a comfortable arrangement with a stable support for an ice-bucket that is about 0.3 meters high (the disposable Styrofoam shipping container for mailing cold or frozen reagents is appropriate) and a low chair for the operator. The ice bucket is placed on the support structure and the chair is drawn up to this unit. You should be comfortable sitting for an extended period in the chair with your elbows resting on your thighs, and with the left hand holding a struggling horseshoe crab positioned above the blood collection tubes propped upright in a bed of ice in the ice bucket.
Pre-chill 4 - 6 of the 50 mL disposable plastic centrifuge tubes with screw closures in the ice bath. The tubes are set upright in the ice ranged around the perimeter of the ice bath container. Just before starting the bleeding operation, remove the caps from the tubes and place caps on adjacent table, adhering to sterile technique.
Remove the cap of a 14 ga needle and use the sturdy forceps to loosen the needle in its sleeve, but do not remove needle from the sleeve. MAINTAIN STERILITY. DO NOT TOUCH ANY PART OF THE NEEDLE WITH THE FINGERS. Place sleeve with needle on an adjacent counter with the exposed butt of the needle elevated from contact with any surface to maintain its sterility.
Moisten a Kimwipe with 70% ethanol.

**Bleeding the animal (specifically for the right-handed operator)**

Holding the animal for bleeding: grasp animal in the left hand with the four fingers in contact with the anterior flange of the prosoma and the thumb grasping the posterior part of the opisthosma at the base of the tail (the terminal bay). The ventral surface of the animal faces the palm of your hand. The telson lies along the base of the thumb and projects over the wrist. Flex the animal gently so that prosoma and opisthosoma are at right angles to one another (Figure 3a).
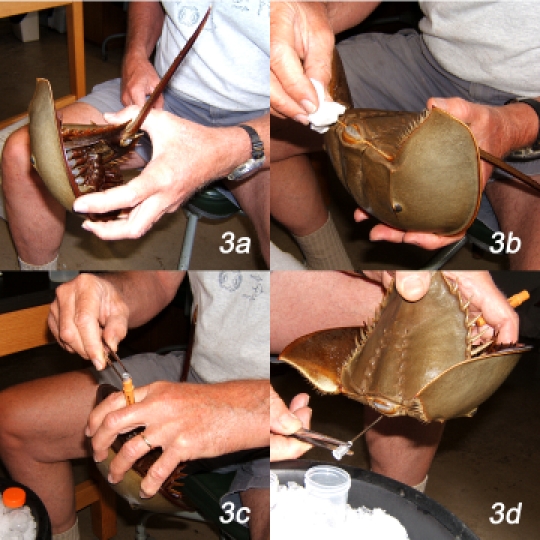
**Figure 3**
Clean the exposed hinge joint connecting prosoma and opisthosoma with 70% ethanol applied with an ethanol-moistened Kimwipe (Figure 3b).
Rotate the left hand such that fingers 2 - 4 are uppermost and the dorsal surface of the animal faces up. With the right hand, insert the 14 ga syringe needle, still in its protective sleeve, between fingers 2 and 3 of the left hand. Take the forceps in the right hand and remove the needle from its protective sleeve by grasping the butt with the forceps (Figure 3c). Rotate the left hand so that dorsal surface of the hand faces to your left, thumb and index finger are up, and the animal is upside down, with its dorsal surface facing the right hand. Position the butt of the needle above the first of the 50 mL collection tubes and orient the animal so that the sharp end of the needle is pointed at the hinge joint and so that the needle shaft is parallel to the dorsal midline (Figure 3d). Insert the sharp end of the needle through the hinge joint into the lumen of the heart, keeping the needle positioned parallel to the dorsal midline and inserted at the midline and with the butt of the needle still positioned above the opening of the collection tube. The needle is inserted about 1 - 2 cm deep into the portion of the heart in the prosoma. As soon as the heart is punctured, blood will flow rapidly (Figure 4a, arrow), so it is important to ensure that the delivery end of the syringe needle is positioned over the mouth of the collection tube to collect that initial surge of blood.
Blood will exit the heart initially in a continuous stream that, after about 30 - 50 mL of blood have been collected, will slow to individual drops (Figure 4b). To improve blood flow, it may be good policy to re-orient the tip of the needle slightly. Tilt the needle up or down, left or right; slide its tip deeper into the heart or pull it to closer to the hinge joint to find the position that optimizes blood flow. Do not compress the animal excessively, because this risks damaging the animal and may cause the extravasated blood to initiate clotting.
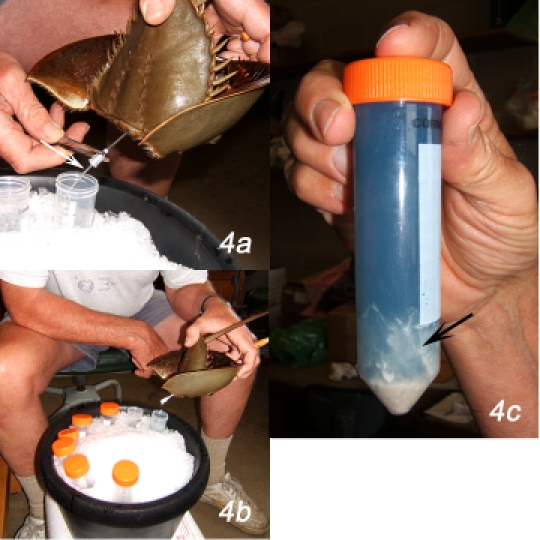
**Figure 4**
As each collection tube fills with blood, rotate the ice bath to bring the next empty tube beneath the delivery end of the needle.
As bleeding slows, you run the risk of having air flow in a retrograde direction into the needle and into the heart. This can occur when the animal straightens out, thereby reducing the blood pressure. Try to prevent this by maintaining the animal in the flexed position or by allowing straightening to occur only slowly.
When the animal stops giving blood, remove the bleeding needle from the heart. Re-cap the collection tubes and centrifuge immediately in the table-top centrifuge, 1000 RPM, 5 min. The blood cells will form compact pellets at the bottoms of the collection tubes. Because all of the components for blood clotting are contained within secretory vesicles of the blood cells, as soon as plasma is separated from cells, the risk of clotting of the plasma is eliminated.
It is imperative to avoid clotting of the extravasated blood. The following measures reduce the risk of clotting: Pre-chilling the animal and maintaining the collection tubes in an ice bath; gentle handling of the animal; reduction of the rapidity with which the blood flows out of the syringe needle; centrifugation of the blood immediately after collection; avoidance of contamination of needles and collection tubes with endotoxin; maintenance of sterile procedure. As was mentioned above, some individual animals have especially irritable blood cells that initiate exocytosis even when these precautions are followed.
After centrifugation, inspect the tubes for signs of blood clotting - strands of cells and gelatinous clot material attached to the walls of the tube, gelatinous material at the top of the clot, or, worse, large amounts of clot with entrapped blood cells in the fluid column (Figure 4c, arrow). The material from these tubes is serum, not plasma, and contains the secreted products of the blood cells.
Transfer the plasma to sterile tubes with the 50 mL sterile serological pipette. Exercise care when pipetting to avoid aspirating any of the cells from the cell pellet in the bottom of the tube. It is best to leave a few mL of plasma above the pellet to avoid disturbing the cell pellet.
The procedure described above is designed to collect large volumes of plasma uncontaminated by the secretory products of the blood cells. To collect large volumes of washed blood cells, collect the blood into 0.1 volumes of 2% Tween-20 dissolved in 3% LPS-free NaCl solution. Centrifuge the cells gently for 10 minutes to establish a loose cell pellet, then wash the cells with several changes of LPS-free 3% NaCl. An extract of the cells that contains the secretory products of the blood cells can be prepared by lysing the washed cells in LPS-free distilled water. This lysate contains a diverse array of proteins and peptides that operate in anti-microbial immune defense ^10,11^ and also contains coagulogen, the zymogen form of the structural protein of the extracellular blood clot, and the collection of proteases that convert coagulogen to coagulin, the form of the protein that polymerizes into the fibrils of the clot ^12^. This extract is similar to the commercially available product, Limulus amebocyte lysate or LAL, which is used to assay for the presence of lipopolysaccharide ^13^. The protease cascade responsible for proteolytic modification of coagulogen to coagulin is activated by LPS ^14^.
Disposition of animals after bleeding. Bleeding conducted by the methods identified above is tolerated well by the animal. When working at the Marine Biological Laboratory at Woods Hole, I return animals to the ocean after bleeding with the expectation that they will survive. If animals are maintained in aquaria distant from the normal range of horseshoe crabs, they can be bled repeatedly once or twice a month. As is described below, animals maintained in captivity require high quality water and frequent feeding to maintain their health. Euthanasia can be accomplished by destruction of the dorsal ganglion, which is situated in the dorsal midline between the eyes.

#### Processing and storage of plasma

Protection from proteases. If the plasma is to be used for protein purification, it should be treated with protease inhibitors immediately after collection. Add phenylmethylsulfonylfluoride (PMSF) to a final concentration of 1 mM. PMSF is kept as a 0.1 M stock solution in DMSO or ethanol. It is quite unstable in water ^15^, so cannot be relied on for lasting protection, but addition immediately after separation of plasma from blood cells will inactivate the small quantities of proteases that might have been released from the cells.
Sterile filtration: sterilize the plasma by ultrafiltration under vacuum using a disposable medium-filtration device fitted with a 0.22 mm pore filter. These filters have a tendency to clog. It is advisable to centrifuge the plasma before filtering to reduce this problem. If large volumes of plasma are processed, it is advisable to pre-filter the plasma first using a Millipore filter with a 1 mm pore size, then with a 0.45 mm pore size Millipore filter.
Plasma can be protected from bacterial contamination either by adding NaN_3_ to a final concentration of 0.2 mg/mL or by sterile filtration. Unsterile plasma should never be stored for periods longer than 2 days without one or the other of these anti-bacterial measures being taken.
Blood clotting. Unlike the mammalian clotting system, the Limulus plasma contains none of the elements for blood clotting. Instead, the entire machinery for clot formation, the structural protein of the clot (coagulogen) and the system of proteases that process coagulogen to render it capable of polymerizing into the insoluble clot, is found in the secretory granules of the blood cells ^16^. So if the blood cells are removed before they have a chance to undergo exocytosis, the plasma is entirely free of the proteins for clotting and will remain fluid indefinitely.
Protein purification. Hemocyanin is present at 40 mg/mL in plasma and is a 48-mer of a 70 kDa subunit. It can be isolated by ultracentrifugation (40,000 X g, 8 h) or gel filtration with a large pore size exclusion resin such as BioGel A5m ^17^. The C-reactive proteins are present at 1 – 5 mg/mL and can be isolated by affinity isolation on phosphorylethanolamine-Sepharose, with elution with a calcium chelator ^18^. The broad-spectrum protease inhibitor, a_2_-macroglogulin, has been purified by gel filtration on Sephacryl S-300 resin after hemocyanin and the C-reactive proteins have been removed ^19^.

**Microscopic examination of the motile blood cells in culture**

Establishment of amebocyte lawns *in vitro*: LPS-free 3% NaCl solution is added to sterile plastic Petri-style culture dishes (1 mL for a 35 mm dish, 2.5 mL for a 60 mm dish, 6 mL for a 90 mL dish). Blood is then obtained by cardiac puncture using the method described above, but with the modification that a 23 gauge, 1 in sterile syringe needle is used instead of the 14 gauge needle. Blood flows drop-by-drop from the 23 gauge needle and 1 drop is collected into the 35 mm dish, 3 drops into the 60 mm dish, and 9 drops into the 100 mm dish. The blood cells are then mixed uniformly in the 3% NaCl solution by gentle swirling, first in a circular pattern, then back-and-forth. The preparation is incubated for 5 min at room T to allow blood cells to attach to the dish surface, then the initial saline is replaced with a buffer of choice^3^.
If it is desired to maintain the substratum-attached blood cells in an undegranulated condition, the initial saline-blood plasma mixture is replaced with fresh LPS-free 3% NaCl. Plasma accelerates the spontaneous degranulation of the blood cells even in the absence of LPS, and its removal stabilizes the undegranulated state of the cells.
If the initial saline is replaced with sterile plasma, the blood cells transform from the ovoid shape of the circulating blood cell (Fig. 5) and flatten onto the substratum, and then initiate degranulation and the formation of a layer of the coagulin extracellular blood clot above the lawn of flattened amebocytes ^20^.
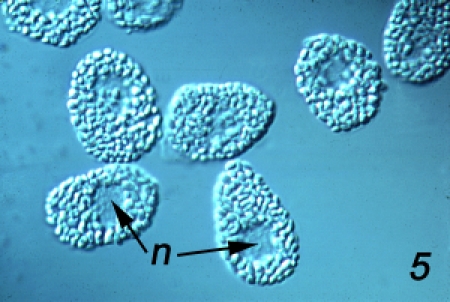
**Figure 5**
Establishment of explant cultures of aggregated amebocytes. When blood is collected under sterile, LPS-free conditions, the blood cells settle out from suspension and aggregate to form a tissue-like mass. A convenient container for this is the glass embryo dish rendered LPS-free by incubation for 4 h at 180^0^C. Blood collected in this container by cardiac puncture using a 19 gauge needle is incubated for 1 – 2 h at room T, then pieces 1 mm square are cut from the amebocyte aggregate with a sterile 18 gauge syringe needle and are sandwiched between two coverglasses or a coverglass and a slide separated by fragments of coverglasses to act as spacers. After about half an hour, amebocytes begin migrating from the periphery of the explant onto the coverglass (Fig. 6a), where they can be viewed with phase contrast or DIC optics ^21^. Initially, the migrating cells are compact with hyaline pseudopods (Fig. 6b (m)) that are extended above the migration substratum, and that then are lowered into contact with the surface and forward motion involves the flow of the granular cytoplasm into the pseudopod ^22^. Later, the cells flatten (Fig. 6b (f)) and initiate degranulation (Fig. 6b (d)) and cease locomotion ^23^. The culture chamber described here allows the perfusion of different experimental agents into the culture chamber to investigate their effects on cell motility and granule exocytosis ^9^. An extended methodological review of amebocyte culture for microscopic examination can be found in ^24^.
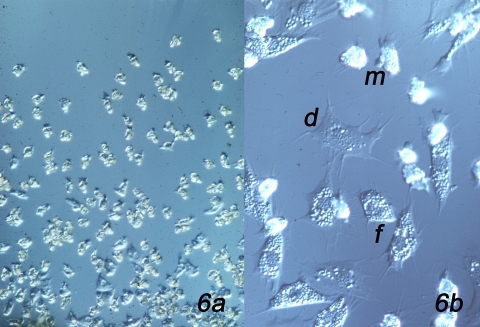
**Figure 6**

#### Maintenance of adult horseshoe crabs in aquaria

Horseshoe crabs require high quality sea water and can be maintained in aquaria fitted with a water purification system that provides particle filtration, aeration, and denitrification of the water^25^. Animals should be fed 2 or 3 times a week on shrimp, lobster, fish, or squid. Feeding is accomplished by removing the animal from the aquarium, placing it on its back, and placing the food on the mouth. If hungry, the animal will soon begin swallowing the food. Depending on the sophistication of the sea water system, the animal should be maintained for an hour or so until it defecates in a separate container of sea water or it can be returned to the aquarium after feeding has begun. For long-term maintenance, the animal should be kept away from light to prevent the growth of green or blue-green algae on the surface of the carapace. Algae erode the carapace and since the adult does not molt, this will cause death to the animal ^26^. In the wild, animals spend much of the time partially buried in sand, but sand or gravel in the aquarium presents a difficulty in maintaining the level of cleanliness important for long-term maintenance.

## Discussion

There are four species of horseshoe crab, *Limulus polyphemus* from the east coast of North America and three species that range from Japan to the Bay of Bengal. The animal is identified as a "living fossil" because anatomically similar forms have been found as fossils from 445 million years ago ^27^. The horseshoe crab represents a long-lived animal, requiring 10 - 13 years to reach maturity and living at least 20 years ^28^. Although it has been subjected to extensive harvesting as bait for the eel and conch fisheries^29^, the American horseshoe crab is still reasonably plentiful and allows the non-destructive collection of 50 mL of blood from a small adult and as much as 400 mL from a large female. Collection of even this much blood can be completed in less than 10 minutes. I know of no other readily available invertebrate that affords so much blood with so little effort.

The importance of the LAL test for endotoxin, an indicator for the presence of Gram-negative bacteria, has stimulated interest in the immune system of the horseshoe crab^13^. The LAL test depends on the activation of the initiating protease of the blood clotting system by LPS^14^, with a read-out of clot formation or of a colorimetric assay for protease activity. The two elements of immunity that have received the greatest attention have been the proteins of the plasma and the several proteins and peptides of the secretory granules of the blood cells. The result has been the elevation of the horseshoe crab to the status of having the best-described immune system for any long-lived invertebrate^11^
